# Mechanical Thrombectomy in Stroke—Retrospective Comparison of Methods: Aspiration vs. Stent Retrievers vs. Combined Method—Is Aspiration the Best Starting Point?

**DOI:** 10.3390/jcm13051477

**Published:** 2024-03-04

**Authors:** Grzegorz Meder, Paweł Żuchowski, Wojciech Skura, Piotr Płeszka, Marta Dura, Piotr Rajewski, Magdalena Nowaczewska, Magdalena Meder, Andrea M Alexandre, Alessandro Pedicelli

**Affiliations:** 1 Department of Interventional Radiology, Jan Biziel University Hospital No. 2, Ujejskiego 75, 85-168 Bydgoszcz, Poland; 2 Department of Radiology, Ludwik Rydygier Collegium Medicum in Bydgoszcz, Nicolaus Copernicus University in Toruń, Jan Biziel University Hospital No. 2, Ujejskiego 75, 85-168 Bydgoszcz, Poland; 3 Department of Rheumatology and Connective Tissue Diseases, Ludwik Rydygier Collegium Medicum in Bydgoszcz, Nicolaus Copernicus University in Toruń, Jan Biziel University Hospital No. 2, Ujejskiego 75, 85-168 Bydgoszcz, Poland; 4 Stroke Intervention Centre, Department of Neurosurgery and Neurology, Jan Biziel University Hospital No. 2, Ujejskiego 75, 85-168 Bydgoszcz, Poland; 5 Department of Neurology, Ludwik Rydygier Collegium Medicum in Bydgoszcz, Nicolaus Copernicus University in Toruń, M. Skłodowskiej—Curie 9, 85-090 Bydgoszcz, Poland; 6 Department of Otolaryngology, Head and Neck Surgery and Laryngological Oncology, Ludwik Rydygier Collegium Medicum in Bydgoszcz, Nicolaus Copernicus University in Toruń, M. Skłodowskiej—Curie 9, 85-090 Bydgoszcz, Poland; 7 Unità Operativa Semplice Autonoma Neuroradiologia Interventistica, Fondazione Policlinico Universitario A. Gemelli IRCCS, Largo A. Gemelli 8, 00168 Roma, Italy

**Keywords:** mechanical thrombectomy, stent retriever, aspiration catheter, combined method, age, ischemic stroke, large vessel occlusion, first-pass effect, IVT, cost

## Abstract

**Background**: There are three main methods of mechanical thrombectomy (MT): using a stent retriever (SR) only (SO), aspiration catheter (AC) only (AO) and the combined method (CM) using both the SR and AC. This paper describes a real-life, single-center experience using SO, AO and CM during 276 consecutive MTs. **Methods**: The primary endpoint was the frequency of first-pass complete (FPE TICI3). The secondary endpoints were final mTICI 2b-3, procedure duration, clinical outcome and the total number of device passes. The third aim of this study was to test the association between the clinical outcomes in patients treated with each method and various factors. **Results**: There was a significant difference (*p* = 0.016) between the groups’ FPE TICI3 rates with 46% mTICI 3 in the AO group, 41% in the CM group and 21% in the SO group. AO resulted in procedure time shortening to a mean duration of 43 min, and the scores were 56 min for CM and 63 min for SO (*p* < 0.0001). There were no significant differences in clinical outcomes or in-hospital mortality. The analysis showed a correlation between good clinical outcomes and the administration of IVT: OR 1.71 (1.03–2.84) *p* = 0.039. Patients ≥66 years old had higher odds of a bad outcome compared to younger patients in general (OR, 1.99 95% CI, 1.17–3.38; *p* = 0.011). FPE TICI3 was associated with good functional outcomes in the whole treated cohort (OR, 1.98; 95% CI, 1.21–3.25; *p* = 0.006). **Conclusions**: In our series, AO proved to be the best starting point in most cases. It demonstrates good technical efficacy regarding FPE, it is fast and clinical outcomes seem to be the least age- and FPE TICI3-dependent. It can be easily converted into the combined method, which had the second-best outcomes in our cohort.

## 1. Introduction

Mechanical thrombectomy (MT) has become the standard of care in stroke caused by large vessel occlusion (LVO) in recent years, and several randomized clinical trials have proven its superiority over the best medical treatment [1,2,3,4]. Blood flow restoration in the occluded vessel can be obtained mechanically through three main methods: using a stent retriever, aspiration catheters and combining both these methods. Many trials comparing those techniques have proven the equality of SO and CM, and many have demonstrated the superiority of CM over AO and SO [5,6,7,8,9]. Of the two recent network meta-analyses, both suggested CM to be superior in terms of technical efficacy with similar safety and clinical outcomes compared to AO and SO [10,11].

This paper describes our real-life experience with a large patient cohort and with different MT methods. We aim to answer the question of which method might be well suited for various patient groups and to identify some of the factors that might have an impact on the final clinical outcome.

## 2. Materials and Methods

### 2.1. Study Population

This retrospective study includes patients, from our database, who underwent endovascular treatment (EVT) for large vessel occlusion between June 2019 and May 2021. During this period, we performed 358 MTs in stroke patients. Patients with anterior circulation stroke, ASPECT score ≥6, who underwent MT either with a stent retriever, aspiration catheter or a stent retriever combined with an aspiration catheter were included in the analysis. Patients with tandem pathologies, artery dissections, and thrombosed stents and cases when the operator switched the technique were excluded (total excluded n = 82). In total, 276 patients were included in the analysis. Out of those patients, 43 were treated with stent retriever only (SO), 106 with aspiration catheters only (AO) and 127 with the combined method (CM). In the entire cohort, a total number of 172 (62.3%) patients received intravenous recombined tissue plasminogen activator (iv-rTPA). There was no difference in the administration of iv-rTPA between the three groups (26 patients (60.4%) in the SO group vs. 85 patients (66.9%) in the AO group vs. 61 patients (57.5%) in the CM group; *p* = 0.326).

Consent for treatment was obtained from the patients in compliance with national guidelines. This study was approved by the bioethical commission: University of Nicolaus Copernicus in Toruń, L. Rydygier Collegium Medicum in Bydgoszcz Medical Ethics Committee [KB 425/2021]. This study was conducted in accordance with the Declaration of Helsinki.

### 2.2. Image Analysis

The angiograms were evaluated by a neuroradiologist with >15 years of experience in interventional radiology and 7 years of experience in mechanical thrombectomy. The images were rated according to the definition of the extended TICI score [12,13]. ASPECTS were calculated using automated software (e-ASPECTS—Brainomix (https://www.brainomix.com/stroke/e-aspects/), Suffolk, UK). Intracranial hemorrhages were graded in compliance with the ECASS III criteria [10].

### 2.3. Thrombectomy Technique

In SO cases, the procedure consisted of introducing a large-internal-diameter guiding catheter (Chaperon, Microvention Inc., Aliso Viejo, CA, USA) or a long neurovascular sheath (Neuron Max, Penumbra Inc., Alameda, CA, USA) into the C1-C2 ICA segment, then navigating with a microcatheter and a microguidewire beyond the occluded segment, placing a selected stent retriever (Solitaire, Medtronic, Minneapolis, MN, USA or Catch, Balt, Montmorency, France or pReset, Phenox, Bochum, Germany or Penumbra3d, Penumbra Inc., Alameda, CA, USA) and extracting the thrombus with the aid of a 50 cc vacuum-locked syringe attached to the long sheath or guiding catheter. In AO cases, the procedure consisted of introducing a long 8F neurovascular sheath (Neuron Max, Penumbra Inc., Alameda, CA, USA) into the C1-C2 segment of ICA through an 8F short introducer placed in the common femoral artery. Then, the largest-available-diameter aspiration catheter (Sofia, Microvention Inc., Aliso Viejo, CA, USA; React, Medtronic, Minneapolis, MN, USA, Ace or Jet, Penumbra Inc., Alameda, CA, USA) with respect to the target vessel size was pushed in, preferably using the so-called “SNAKE technique” to engage with the proximal end of the clot, and then the thrombus was aspirated with the aid of a 50 cc vacuum-locked syringe attached to the aspiration catheter [14,15]. In cases of tortuous ICA or difficulties when passing the origin of the ophthalmic or other intracranial ICA branches, a microcatheter or a looped guidewire was used to facilitate aspiration catheter movement.

In cases when the CM was used, the procedure consisted of introducing a long 8F neurovascular sheath (Neuron Max, Penumbra Inc., Alameda, CA, USA) into the C1-C2 segment of ICA through an 8F short introducer placed in the common femoral artery. Then, a system consisting of a large-bore aspiration catheter (Sofia, Microvention Inc. Aliso Viejo, CA, USA; React, Medtronic, Minneapolis, MN, USA, Ace/Jet, Penumbra Inc. Alameda, CA, USA; Embovac, Cerenovus, Fremont, CA, USA), a microcatheter and microguidewire were introduced, with the microcatheter passing through the thrombus. After placing a selected stent retriever (Embotrap, Cerenovus, Fremont, CA, USA or Solitaire, Medtronic Minneapolis, MN, USA or Catch, Balt, Montmorency, France or pReset, Phenox, Bochum, Germany or Penumbra3d, Penumbra Inc., Alameda, CA, USA or Neva, Vesalio, Nashville, TN, USA), the aspiration catheter was moved up to engage with the front of the thrombus, then the microcatheter was removed to increase the working lumen of the system. The final step consisted of pinching the clot by partially pulling the SR inside the AC and removing the whole system (SR + AC) from the long sheath with the aid of the aspiration of a 50 cc syringe or mechanical pump (Penumbra Inc., Alameda, CA, USA). In ideal conditions, this procedure is identical to the SAVE technique [8,9]. In some cases such as kinked, tortuous ICA and difficult navigation, the technique was modified and usually only the SR was removed, leaving the AC in place for potential further maneuvers. In M2 occlusions, unless the occluded branch was large enough to accommodate the chosen AC, usually, the AC was pushed only to the end of the M1 segment, and the SR with a clot was then removed as described above. A list of endovascular equipment used in the analyzed cohort is presented in Supplementary Table S1.

### 2.4. Aim of the Study

The aim of this single-center cohort study is to compare the three main mechanical thrombectomy methods. The assignment of the method of treatment was at the discretion of the two performing physicians (G.M. or W.S.).

The first aim of this study was to establish the technical efficacy of the three main MT methods. In order to test it, we assessed the following: frequency of perfect or near-perfect reperfusion (defined as TICI 3 or TICI 2c-3)after the first pass, the procedure success defined as final mTICI score 2b-3, the mean duration of the procedure, the mean number of device passes and the hemorrhagic complications.

The second aim of this study was to establish the clinical efficacy of the three main MT methods. To test it, we assessed the following: ∂ NIHSS defined as the difference between NIHSS at patients’ admission and discharge (excluding fatal outcomes), in-hospital mortality and 90-day modified Rankin Scale (mRS).

The third aim of this study was to test the association between the good (90-day mRS 0-2) and bad (90-day mRS 3-6) clinical outcomes in patients treated with each method and factors such as age, procedure duration, administration of IVT and FPE TICI 3.

### 2.5. Statistical Analysis

In the statistical analysis, a detailed examination of the data collected for this study was conducted. Continuous data were presented using various measures, including mean with standard deviation, median and IQR. The normality of the continuous data distribution was assessed using the Shapiro–Wilk test. To compare differences between groups, a one-way ANOVA test was used for continuous data, and for categorical variables, the chi-square test was applied. Additionally, odds ratios along with a 95% confidence interval were calculated for relevant variables. Statistical significance was set at *p* < 0.05. Calculations were performed using the MedCalc^®^ Statistical Software version 22.013 (MedCalc Software Ltd., Ostend, Belgium; https://www.medcalc.org (accessed on 3 August 2023) program.

## 3. Results

### Study Population

Two hundred and seventy-six patients were included in this study. Baseline characteristics of the study population are described in Table 1. Out of the 276 patients included, 43 were treated with a stent retriever only, 106 patients with an aspiration catheter only and 127 patients with a stent retriever and aspiration catheter. The characteristics of the study population are presented in Table 1.

There were no statistical differences in the population subgroups regarding the occlusion site, age, sex, NIHSS at admission, comorbidities, ivTPA administration and time from symptom onset to thrombectomy. The only statistically significant difference was in the SO group, which had a slightly better ASPECTS score at admission.

The results of the technical and clinical efficacy analysis are presented in Table 2. There was a significant difference (*p* = 0.016) between the groups regarding first-pass success rate with 46% mTICI 3 in the AO group, 41% in the CM group and 21% in the SO group. With regard to near-complete recanalization (mTICI 2c-3), those differences were even more prominent, achieving 58% in the AO group vs. 50% in the CM group and 23% in the SO group, with *p <* 0.001. The complete procedure success, described as final mTICI 2b or more, was most frequent (95%) in the AO group, then in the CM and SO groups, it was 90% and 88%, respectively, yet the difference was not statistically significant (*p* = 0.62). The aspiration-only approach required the lowest (1.6 ± 1.1) mean number of passes per procedure, while the combined method and stent retriever-only method needed 1.9 ± 1.4 and 2.1 ± 1.0, respectively; however, those differences were not statistically significant (*p* = 0.097). Regarding the mean duration of the procedure, the analysis showed, with a high level of statistical significance (*p <* 0.0001), that using AO resulted in procedure time shortening to a mean duration of 43 min; the scores were 56 min for the CM and 63 min for the SO groups. In terms of clinical outcomes such as 90-day mRS; ∂-NIHSS; and complications such as all intracranial hemorrhages, sICH and in-hospital mortality, there were no significant differences between the analyzed groups.

The 90-day mRS outcomes split into groups with mRS 0-2 (good outcomes) and mRS 3-6 (bad outcomes) are summarized in Table 3. While we observed differences between groups, with AO yielding the lowest percentage of good outcomes vs. SO or CM, those differences were not statistically significant.

Univariate analyses of potential factors associated with clinical outcomes are presented in Table 4, Table 5, Table 6, Table 7 and Table 8. We observed (Table 4) significant differences in mean procedure duration times between groups with AO being the shortest procedure in all patients regardless of the clinical outcome. A significant difference (*p* = 0.007) in the mean duration of the CM procedures in patients with good outcomes (mean 49.1 min) vs. in patients with bad outcomes was also observed (62.0 min).

With regard to the mean age of patients with good or bad outcomes (Table 5), we found no statistically significant differences in patients treated with aspiration only, whereas patients with bad outcomes treated with stent retrievers only or with the combined method were significantly older then patients with good outcomes treated with the same methods (77.3 vs. 68.7, *p* = 0.009 for SO and 76.7 vs. 66.1, *p* = 0.00001 for CM).

**Table 5:** The 90-day mRS split into groups with mRS 0-2 (good outcomes) and mRS 3-6 (bad outcomes) in relation to the mean age of patients.

	mRS 0-2 Age [Mean]	mRS 3-6 Age [Mean]	*p*
AO	72.1 ± 12.9	72.4 ± 11.4	0.921
SO	68.7 ± 11.5	77.3 ± 8.8	0.009
CM	66.1 ± 17.4	76.7 ± 12.7	0.00001

The analysis of the association between intravenous thrombolysis and clinical outcomes (Table 6) revealed a significant correlation between good clinical outcomes and administration of IVT in general: OR 1.71 (1.03–2.84), *p* = 0.039 for the total number of 276 patients. This correlation was also observed in AO- and SO-treated groups but was statistically significant only in AO-treated patients, with OR 2.45 (1.05–5.73), *p* = 0.038. There seems to be no correlation between the administration of IVT and clinical outcomes in patients treated with the combined method, with OR 1.03 (0.48–2.17), *p* = 0.943.

**Table 6:** The 90-day mRS split into groups with mRS 0-2 (good outcomes) and mRS 3-6 (bad outcomes) in relation to IV thrombolysis.

Method	mRS-90	IVT—Yes	IVT—No	Odds Ratio (95% CI), *p*
AO	mRS 0-2	27 (53%)	11 (24%)	2.45 (1.05–5.73) *p* = 0.038
	mRS 3+	34 (47%)	34 (76%)	
SO	mRS 0-2	14 (54%)	5 (29%)	2.80 (0.77–10.25) *p* = 0.120
	mRS 3+	12 (46%)	12 (71%)	
CM	mRS 0-2	37 (44%)	18 (43%)	1.03 (0.48–2.17) *p* = 0.943
	mRS 3+	48 (56%)	24 (57%)	
Total	mRS 0-2	78 (45%)	34 (33%)	1.71 (1.03–2.84) *p* = 0.039
	mRS 3+	94 (55%)	70 (67%)	

Testing the association between the clinical outcomes of various methods of treatment in patients aged ≤65 and ≥66 years revealed that patients ≥66 years old had higher odds of bad outcomes compared to younger patients in general (OR, 1.99 95% CI, 1.17–3.38; *p* = 0.011), particularly in patients treated with CM (OR, 3.09 95% CI, 1.35–7.04; *p* = 0.007).

**Table 7:** The 90-day mRS split into groups with mRS 0-2 (good outcomes) and mRS 3-6 (bad outcomes) in groups of patients ≤65 years and ≥66 years.

Method	mRS 90	≤65 Years	≥66 Years	Odds Ratio (95% CI), *p*
AO	mRS 0-2	11 (46%)	27 (33%)	1.72 (0.68–4.35) *p* = 0.249
	mRS 3+	13 (54%)	55 (67%)	
SO	mRS 0-2	6 (60%)	13 (39%)	2.31 (0.54–9.79) *p* = 0.257
	mRS 3+	4 (40%)	20 (61%)	
CM	mRS 0-2	21 (64%)	34 (36%)	3.09 (1.35–7.04) *p* = 0.007
	mRS 3+	12 (36%)	60 (64%)	
Total	mRS 0-2	38 (57%)	74 (35%)	1.99 (1.17–3.38) *p* = 0.011
	mRS 3+	29 (43%)	135 (65%)	

Successful recanalization defined as TICI 3 after the first attempt was associated with good functional outcomes (Table 8) in the whole treated cohort (OR, 1.98; 95% CI, 1.21–3.25; *p* = 0.006) but particularly in patients treated with stent retrievers only (OR, 5.65, 95% CI, 1.01–3.47; *p* = 0.048) or treated with the combined method (OR, 3.13, 95% CI, 1.50–6.55; *p* = 0.002). The first-pass effect was not observed in the subgroup of patients treated with aspiration only.

**Table 8:** The 90-day mRS split into groups with mRS 0-2 (good outcomes) and mRS 3-6 (bad outcomes) in relation to the first-pass effect (FP TICI 3).

Method	mRS-90	FP TICI 3: Yes	FP TICI 3: No	Odds Ratio (95% CI), *p*
AO	mRS 0-2	18 (37%)	20 (35%)	1.07 (0.48–2.38) *p* = 0.860
	mRS 3+	31 (63%)	37 (65%)	
SO	mRS 0-2	7 (78%)	13 (38%)	5.65 (1.01–3.47) *p* = 0.048
	mRS 3+	2 (22%)	21 (62%)	
CM	mRS 0-2	31 (60%)	24 (32%)	3.13 (1.50–6.55) *p* = 0.002
	mRS 3+	21 (40%)	51 (68%)	
Total	mRS 0-2	56 (51%)	57 (34%)	1.98 (1.21–3.25) *p* = 0.006
	mRS 3+	54 (49%)	109 (66%)	

## 4. Discussion

In recent years, mechanical thrombectomy has become the standard treatment for stroke caused by large vessel occlusion [1,2,3,6,16,17,18].

Several methods of clot extraction have been described so far, of which two key ones include using aspiration catheters or stent retrievers. It has been proven that both methods have comparable efficacy [7]. As these devices differ regarding their intra-arterial navigability and they engage with the clot in different ways, it was suggested that combining these two methods helps to overcome their weaknesses and improve efficacy and patient outcomes [6]. Different techniques combining stent retrievers with aspiration catheters such as SAVE, Solumbra or ARTS show high reperfusion efficacy with good clinical outcomes [8,9,19,20,21,22,23,24]. In this paper, we analyzed a cohort of 276 consecutive patients who underwent EVT for anterior circulation LVO stroke in our center.

Fast and complete reperfusion is crucial for favorable outcomes [19,25,26,27,28,29,30]. The duration of the procedure may be affected by factors such as the patient’s anatomy, technical complexity, and efficacy of the procedure in achieving full reperfusion. As the first part (gaining access to ICA) of each analyzed procedure in our cohort consisted of the same steps described in the “thrombectomy technique” Section 2.3, efficacy and technical complexity should play a major role in the duration of the EVT. AO proved to be the most efficient method in achieving first-pass TICI 3 and first-pass TICI 2c-3 results, followed closely by more complex CM. SO had the lowest first-pass success rate. The factor that might play a role in SO achieving less favorable results was not using balloon guide catheters. Several papers stressed that the flow arrest obtained when using BGCs greatly improves SO technical results, but it is still not used in the majority of cases [31,32,33,34,35]. Repeating MT maneuvers in SO at the end leads to insignificant differences in the final TICI score but at the expense of a higher mean number of passes and significant prolongation of the procedure. Many subsequent manipulations may, however, potentially negatively affect clinical outcomes, leading to complications such as parenchymal bleeding, embolization to new territories, distal embolizations and arterial endothelial injury, which may result, in some cases, in early reocclusion [28,36,37,38,39,40,41].

Clinical results of EVT in our study did not seem to directly reflect clear differences in angiographic and technical results in the whole cohort. We did not find statistically significant differences in 90-day mRS, dNIHSS and in-hospital mortality, which may be understandable as there are more factors that may affect clinical outcomes. Recent meta-analyses comparing direct aspiration vs. stent retrievers vs. the combined method also failed to prove the superiority of one method of MT over another [42,43].

Dividing patients into two subgroups with regard to 90-day mRS score—good (mRS 0-2) and bad (mRS 3-6) outcomes—and analyzing factors such as procedure duration, the patient’s age, administration of IVT and FP TICI 3 revealed that the procedure duration was significantly (*p* = 0.007) associated with bad outcomes but only in patients treated with CM (62.0 ± 29.1 vs. 49.1 ± 21.7). There was statistically a significant correlation between bad outcomes and older age but only in patients treated with SO (77.3 ± 8.8 vs. 68.7 ± 11.5; *p* = 0.009) and CM (76.7 ± 12.7 vs. 66.1 ± 17.4; *p* = 0.00001). In the whole cohort, patients >65 years old had 1.99 times higher odds (*p* = 0.011) of having bad outcomes after MT regardless of the method, but mostly in cases treated with CM (OR 3.09, *p* = 0.007). Procedure duration and older age have been recognized previously as independent predictors of bad outcomes of MT [44,45].

With aging, degenerative changes in brain vessels have been described as endothelial dysfunction, vessel wall recomposition leading to decreased contractile function, and reduced distensibility and compliance [46]. The results of our study suggest that aspiration seems to be the least aggressive method of MT that can achieve similar good results regardless of patient age and procedure duration. This may be logical because if aspiration is applied properly on the clot itself, and not so much on the vessel wall, it leads to the smallest possible endothelial injury, which has been demonstrated in experimental studies before [38,39]. Partially dissolving the clot, changing its structure and diminishing its size with IVT help to remove it. IVT has been proven to be a beneficial factor in MT [47]. In our treated cohort, IVT played a major role in achieving good outcomes (OR 1.71) and differences were significant in cases treated with AO (OR 2.45).

Analyzing the association of FPTICI3 and outcomes revealed that in cases of successful FPTICI3, the odds ratio of good outcome was higher in the whole cohort (1.98), particularly in cases treated with SO (OR 5.65) and CM (OR 3.13). It did not differ in cases treated with AO (OR 1.07). The importance of FPTICI3 was described earlier and we are not sure why FPTICI3 did not seem to be associated with outcomes in cases of AO. Further investigation on this phenomenon is required but our suggestion is that it is easier on the vessel walls and, additionally, it may be less prone to distal embolizations or embolizations to new territories, especially when the SNAKE technique is used, which assumes that no device is passing the thrombus, possibly causing its fragmentation.

Regardless of clinical and technical efficacy, cost effectiveness may be one more reason to consider AO to be the method of first choice. Despite medical companies having different pricing strategies in various markets, AO has been proven to be the least expensive method [48,49]. This was also true in our cohort. Taking the mean price of direct costs of endovascular equipment needed to perform the MT procedure, the price for AO was USD 2790, USD 3925 for SO and USD 4950 for CM.

The limitations of the presented study are its retrospective and single-center design and the fact that the MT technique used was chosen by the operator. On the other hand, we present our real-world experience from a single comprehensive stroke center that covers a population of approximately 2 million people. All the procedures were performed by neuroradiologists with more than 5 years’ experience in mechanical thrombectomy, each performing >100 MTs per year.

## 5. Conclusions

Taking the above results into account, it may be difficult to recommend the best method of MT for all patients, but it seems that AO might be the best starting point in most cases. It proved to have good technical efficacy regarding FPE, it is fast and its clinical results seem to be the least age- and even FPTICI3-dependent. It can be easily converted into the combined method, which had the second-best results in our cohort. The good results of AO may be affected by the lack of IVT, but this can be overcome after conversion to CM, which proved to be IVT-independent.

## Figures and Tables

**Table 1:** Baseline characteristics of the study population.

Parameter	Overall (n = 276)	SO (n = 43)	CM (n = 127)	AO (n = 106)	*p*-Value
Site of occlusion					
Carotid	54	6	28	23	0.286
M1	144	22	62	60	
M2	69	12	20	37	
Age (yrs ± SD)	72.4 *±* 1364	73.5 *±* 10.9	72.1 *±* 15.7	72.3 *±* 11.9	0.838
Male sex	120 (44%)	18 (42%)	55 (43%)	47 (44%)	0.961
Atrial fibrillation/ICD/PM	127 (46%)	22 (51%)	60 (47%)	45 (42%)	0.584
Arterial hypertension	213 (77%)	36 (84%)	96 (76%)	81 (76%)	0.07
Coronary heart disease	68 (25%)	12 (28%)	33 (26%)	23 (22%)	0.649
Dyslipidemia	99 (36%)	20 (47%)	46 (36%)	33 (31%)	0.206
ASPECTS median, (IQR)	7 (6–8)	8 (7–9)	7 (6–8)	7 (6–7)	<0.001
NIHSS admission median, (IQR)	16 (13–20)	17 (13–21)	15 (12–19)	15 (14–20)	0.222
Symptom onset to thrombectomy in minutes (mean ± SD)	268 *±* 79	261 *±* 79	268 *±* 76	272 *±* 84	0.755
ivTPa	172 (62.3%)	26 (60.4%)	85 (66.9%)	61 (57.5%)	0.326

**Table 2:** Analysis of the technical and clinical efficacies of the MT methods used in this study.

Goal	SO	CM	AO	*p*-Value
First pass mTICI 3	9 (21%)	52 (41%)	49 (46%)	0.016
First pass mTICI 2c-3	10 (23%)	63 (50%)	61 (58%)	<0.001
Procedure success mTICI 2b-3	38 (88%)	114 (90%)	101 (95%)	0.62
Mean number of passes needed	2.1 ± 1.0	1.9 ± 1.4	1.6 ± 1.1	0.097
Mean duration of the procedure (minutes ± SD)	63 ± 32	56 ± 27	43 ± 23	<0.0001
90-day mRS, median (IQR)	3 (1-6)	3 (1-6)	4 (1-6)	0.83
∂ NIHSS (± SD) (excluding deaths)	7.5 ± 4.8	6.9 ± 5.9	7.1 ± 6.7	0.92
In-hospital mortality	12 (28%)	34 (27%)	34 (32%)	0.66
SICH (%)	2 (5%)	11 (9%)	9 (8%)	0.681
ICH + SAH	7 (16%)	18 (14%)	11 (10%)	0.548

**Table 3:** The 90-day mRS split into groups with mRS 0-2 (good outcomes) and mRS 3-6 (bad outcomes).

	mRS-90 (0-2) No (%) of Patients	mRS-90 (3-6) No (%) of Patients	*p*-Value
AO	38 (35.8)	68 (64.2)	0.448
SO	19 (44.1)	24 (55.9)	
CM	55 (43.3)	72 (56.7)	

**Table 4:** The 90-day mRS split into groups with mRS 0-2 (good outcomes) and mRS 3-6 (bad outcomes) in relation to the duration of the procedure.

	mRS 0-2, Procedure Duration [Min]	mRS 3-6, Procedure Duration [Min]	*p*-Value
AO	43.1 ± 31.1	43.5 ± 24.3	0.931
SO	61.1 ± 31.1	65.8 ± 34.2	0.645
CM	49.1 ± 21.7	62.0 ± 29.1	0.007
*p*-value	0.024	0.0001	

## Data Availability

The data presented in this study are available upon a reasonable request from the corresponding author. The data are not publicly available due to privacy restrictions.

## Supplementary Materials

The following supporting information can be downloaded at: https://www.mdpi.com/article/10.3390/jcm13051477/s1, Table S1. The list of main endovascular equipment used in the analyzed cohort.
