# Supplementary material for: Mechanical Thrombectomy in Stroke—Retrospective Comparison of Methods: Aspiration vs. Stent Retrievers vs. Combined Method—Is Aspiration the Best Starting Point?
Source: J Clin Med. 2024 Mar 4;13(5):1477. doi: 10.3390/jcm13051477 (PMC10934276; doi:10.3390/jcm13051477)
Supplement: Supplementary file 1 [file jcm-13-01477-s001.zip › jcm-2853856-supplementary.pdf]

Supplementary Table S1. The list of main endovascular equipment used in the analyzed cohort

Equipment	Method / Cases			
	SO	CM	AO	overall
Nauron MAX, Penumbra USA	16	127	106	249
Chaperon, Microvention USA	27	-	-	27
sum	**43**	**127**	**106**	**276**
Sofia, Microvention USA	-	68	85	153
React, Medtronic USA	-	4	2	6
Embovac, Cerenovus USA	-	1	-	1
Ace / Jet, Penumbra USA	-	54	19	73
sum	-	**127**	**106**	
Embotrap, Cerenovus USA	-	25	-	25
Solitaire, Medtronic USA	2	17	-	19
pReset, Phenox Germany	33	14	-	47
Catch, Balt France	6	40	-	46
NeVa, Vesalio USA	-	17	-	17
Penumbra 3d, Penumbra USA	2	14	-	16
sum	**43**	**127**	-	
